# Synthesis of metformin-derived fluorescent quantum dots: uptake, cytotoxicity, and inhibition in human breast cancer cells through autophagy pathway

**DOI:** 10.1186/s13036-024-00433-4

**Published:** 2024-06-25

**Authors:** Ali Akbari, Mohadeseh Nemati, Zohreh Mehri Lighvan, Fereshteh Nazari Khanamiri, Jafar Rezaie, Yousef Rasmi

**Affiliations:** 1 Solid Tumor Research Center, Cellular and Molecular Research Medicine Institute, Urmia University of Medical Sciences, P.O. BoX: 1138, Shafa St, Ershad Blvd, Urmia, 57147 Iran; 2 Department of Biochemistry, Faculty of Medicine, Urmia University of Medical Sciences, Urmia, Iran; 3 Department of Polymer Processing, Iran Polymer and Petrochemical Institute, P.O. Box 14965-115, Tehran, Iran

**Keywords:** Carbon dots, Hydrothermal, Metformin, Cancer; Autophagy

## Abstract

**Background:**

Breast cancer remains a challenge for physicians. Metformin, an antidiabetic drug, show promising anticancer properties against cancers. An emerging quantum dot (QD) material improves therapeutic agents’ anticancer and imaging properties. QD are nano-sized particles with extreme application in nanotechnology captured by cells and accumulated inside cells, suggesting bioimaging and effective anticancer outcomes. In this study, a simple one-pot hydrothermal method was used to synthesize fluorescent metformin-derived carbon dots (M-CDs) and then investigated the cytotoxic effects and imaging features on two human breast cancer cell lines including, MCF-7 and MDA-MB-231 cells.

**Results:**

Results showed that M-CDs profoundly decreased the viability of both cancer cells. IC50 values showed that M-CDs were more cytotoxic than metformin either 24–48 h post-treatment. Cancer cells uptake M-CDs successfully, which causes morphological changes in cells and increased levels of intracellular ROS. The number of Oil Red O-positive cells and the expression of caspase-3 protein were increased in M-CDs treated cells. Authophagic factors including, AMPK, mTOR, and P62 were down-regulated, while p-AMPK, Becline-1, LC3 I, and LC3 II were up-regulated in M-CDs treated cells. Finally, M-CDs caused a decrease in the wound healing rate of cells.

**Conclusions:**

For the first, M-CDs were synthesized by simple one-pot hydrothermal treatment without further purification. M-CDs inhibited both breast cancer cells through modulating autophagy signalling.

**Graphical Abstract:**

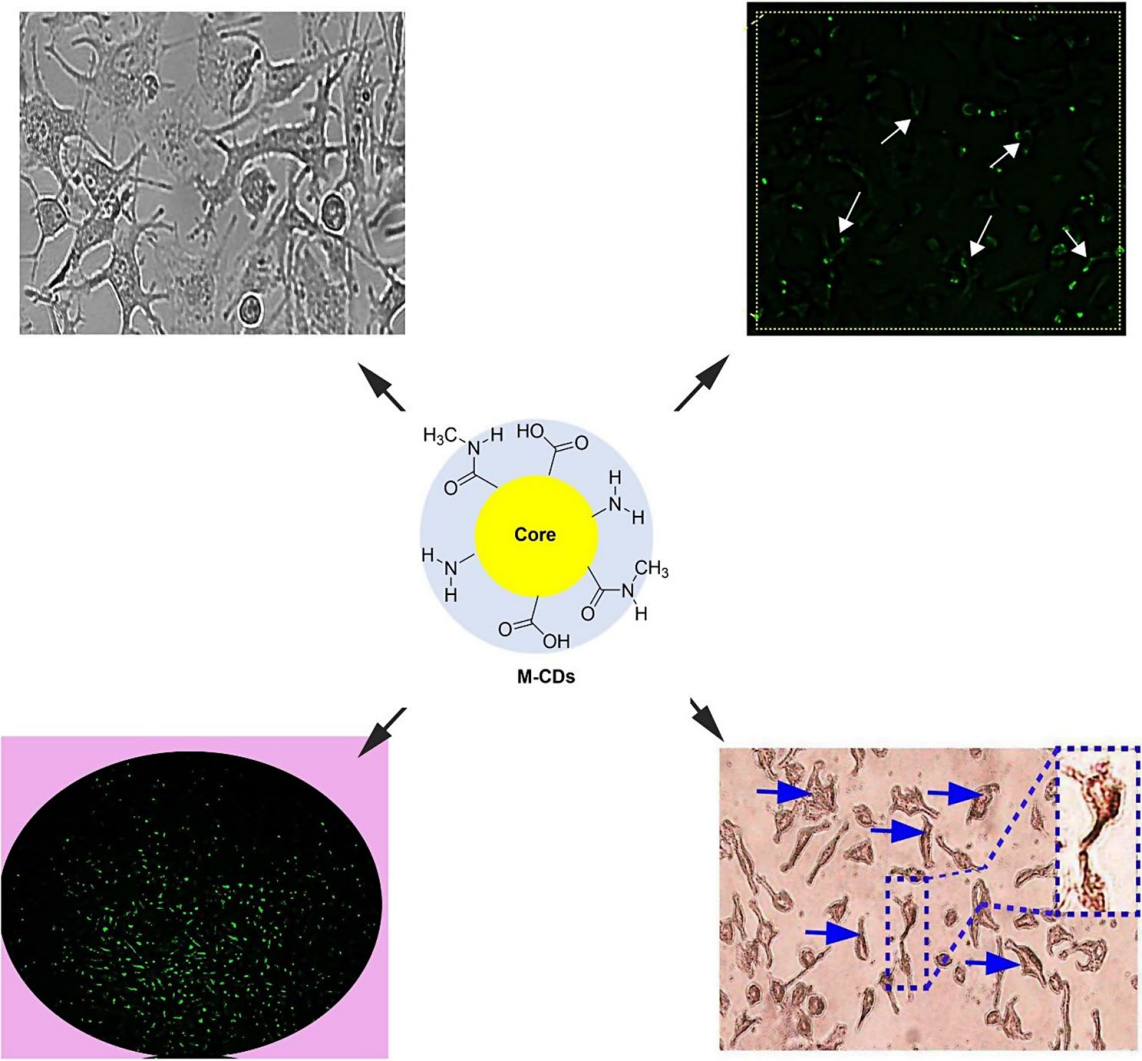

## Background

Breast cancer, the main cause of cancer-related mortality worldwide, has become a challenge for clinicians due to the resistance and insensitivity of tumor cells to traditional therapies [1]. Breast cancer is the fifth leading reason of cancer-related mortality global [2]. In recent years, the application of nanotechnology in oncology has led to the innovation of nano-sized therapeutic agents for efficient and targeted therapy against metastatic tumor cells [3]. Carbon dots (CDs), carbon nanoparticles with less than 10 nm in size, are attracting considerable interest in biomedical applications due to their unique properties including, high biocompatibility and loading efficiency, unique photo-stability in biological systems, photoluminescence, and incredible surface functionality [4, 5]. CDs are useful for tracking and imaging tumor masses, early diagnosis, and cancer treatment. After being injected into the body, CDs can bind to biomolecules such as peptides, antibodies, proteins or DNA strands, therefore following ultraviolet radiation, they can be visible, making them suitable tools for both cancer imaging and therapy [6, 7]. These particles can be synthesized from various organic and inorganic precursors for biomedical applications using bottom-up or top-down approaches [8]. Metformin, N,N-dimethylbiguanide, is used as a blood sugar reducer medication for the treatment of type 2 diabetes (non-insulin-dependent diabetes) [9]. The main molecular mechanism is still not fully understood, however, studies have shown that inhibition of complex I in the mitochondrial membrane, induction of protein kinase AMPK, and suppression of mitochondrial glycerophosphate dehydrogenase are among the pathways targeted by metformin in diabetic conditions [10, 11]. Researchers have recently seen metformin can inhibit cancer cells. Targeting the AMPK/mTOR pathway with metformin may result in decreased protein synthesis and cell growth, which may correlate with the antitumor activity of metformin in cell growth and apoptosis pathways [11, 12]. This pathway can regulate the autophagy pathway in tumor cells [13]. In general, autophagy is a major cellular process for tumor cell growth and metastasis [14]. More importantly, recent evidence indicated that CDs synthesized from metformin could cross from the blood-brain barrier in zebrafish and also target mitochondria in cells in vitro models [15]. Mitochondria is the main organelle in cells with pivotal roles in cellular processes including cell proliferation [16]. This evidence has given the impetus to explore the role of metformin and CDs-derived from metformin (M-CDs) in the regulation of tumor cell proliferation. In this study, we first prepared M-CDs and then hypothesized whether M-CDs induce inhibitory effects on human breast cancer cells with a focus on the autophagy pathway.

## Experimental

### Instrumentations

Fourier transform infrared (FTIR) spectrum was obtained in the spectral range of 500–4000 cm^− 1^ on a Bruker 113 V FTIR spectrometer using KBr pellets. UV-Vis adsorption spectra and photoluminescence (PL) experiments were recorded using a Shimadzu UV-1800 UV–visible spectrophotometer and an F-7000 fluorescence spectrofluorimeter (Hitachi High Technologies, Japan), respectively. transmission electron microscopy (TEM) images of the synthesized M-CDs were recorded on a Philips EM 208 S microscope. Atomic force microscopy (AFM) were collected using a Nanowizard 2 Scanning Probe Microscope.

### Synthesis of M-CDs

The M-CDs were prepared using a simple one-step pyrolysis method. Typically, starting materials, CA (1 g) and metformin hydrochloride (1.16 g), with 1:1 M ratio were dissolved in 50 ml deionized (DI) water in a glass beaker and stirred for 20 min to obtain a transparent solution. Then, the mixture was heated in a Teflon-lined autoclave chamber in the oven at 200 °C for 12 h. Upon completion of the hydrothermal reaction, the successful formation of M-CDs could be observed by changing the color of the mixture solution to dark brown. The final product was obtained without further separation and purification methods.

### Cell lines and cell culture

Two human breast cancer cell lines including MCF-7 and MDA-MB-231 cells were obtained from the Iranian Biological Resource Center (IBRC), Iran. Cells were cultured in RPMI media containing 10% fetal bovine serum (FBS) 0.1 U/L penicillin and 0.1 mg/ml streptomycin. For each experiment, cells were seeded on proper tissue culture plates with a necessary number of cells following a maximum 5th passage. Cells were kept in the humidified incubator (5% CO2) at 37 °C. All cell culture supplements and materials were obtained from Gibco.

### Cell viability

Human breast cancer cells were cultured onto 96-well plates at a density of 7 × 10 ^3^ cells/well and cultured for 24 h. The metformin and M-CDs were diluted to a serial of concentrations (0 ppm, 5 ppm, 10 ppm, 20 ppm, 40 ppm) in RPMI supplemented with 2% FBS, then poured into the corresponding wells. Afterwards, the cells were kept for 24–48 h before 20 µL of MTT reagent (3-[4,5-dimethylthiazol-2yl]-2,5-diphenyl-tetrazolium bromide) (Sigma) was added to a well. After 4 h incubation in a humidified incubator, dimethyl sulfoxide (DMSO) was replaced, and subsequently, the relative cell viability of treated cells was calculated by the measurement of the optical density at 570 nm against control cells. For downstream experiments, we use the IC50 value of either metformin or M-CDs.

### Cellular uptake

To measure the intracellular uptake of M-CDs, 5 × 10^4^ cells/well were seeded onto a 6-well slide chamber (SPL) and kept for 24 h. After incubation with M-CDs for 4 h, slides were washed with PBS thrice, and then the cytoplasmic distribution of M-CDs was visualized using immunofluorescence microscopy (echo LAB microscopy with NFP-1 Fluorescence Microscope Power Supplier Lab). All images were captured with a supplementary charged-coupled device (CCD) camera (TrueChrome II).

### Cell morphological assay

A total of 3 × 10^4^/well were coated on 24-well plates for 24 h, then cells were adopted into the treatment protocol. Next, cells were washed with PBS twice to remove cell derbies. Images from cells were taken using an inverted microscopy (IM-3/ OPTIKA) equipped with CCD camera (TrueChrome II)and compared with control cells visually.

### Oil Red O staining

For the Oil Red O staining, 3 × 10^4^ cells were plated onto per well of the 24-well plates overnight. After the treatment protocol, cells were washed with PBS twice and received methanol for 10 min at RT, and then paraformaldehyde solution (PFA, 4%) was added to fix cells for 30 min at RT. Next, the Oil Red O staining solution (0.1%) was replaced with PFA 4% for 30 min. Finally, cells were washed with PBS three times, and then images were taken by an invert microscopy (IM-3/ OPTIKA)equipped with CCD camera (TrueChrome II).

### Intracellular ROS production

In brief, 5 × 10^4^ cells were cultured in each well of a 12-well plate overnight. Following the treatment protocol, a fluorometric intracellular ROS kit (D6883, Sigma-Aldrich) was used to measure the production of intracellular ROS. According to the kit’s protocol, DCDA reagent was added to cells for 20 min at RT, and then cells were washed and observed for ROS using fluorescence microscopy equipped with CCD camera (echo LAB microscopy with NFP-1 Fluorescence Microscope Power Supplier Lab).

### Western blotting

Briefly, 3 × 10^5^ cells/well were seeded onto 6-well cell-culture plates for 24 h, and after adopting to the treatment protocol, cells were lysed using RIPA buffer (Sigma-Aldrich). Then, 10% SDS polyacrylamide gel electrophoresis gels (Sigma-Alderich) were used to separate cell lysates and then transferred to polyvinylidene difluoride membranes. Subsequent, the membranes were co-incubated with the below primary antibodies overnight at 4°C: anti-AMPK (sc-74,461, 1:1000), anti-LCT3B (877-616-CELL (2355), 1:1000), anti-Beclin-1 (sc-48,341, 1:1000), anti-mTOR (sc-517,464, 1:1000), anti-P62 (sc-10,117, 1:1000), anti-p-AMPK (sc-33,524, 1:1000), anti-caspase-3 (sc-7272, 1:1000), anti-β-Actin (sc-47,778, 1:1000). After washing with TBST, the samples were then incubated with the matching secondary antibodies mouse anti-rabbit IgG-HRP (sc-2357) and m-IgGκ BP-HRP (sc-516,102). The protein bands were then visualized using enhanced chemiluminescence reagents (Sigma-Alderich) and analyzed using Image J software (ver. 1.44p).

### In vitro scratch assay

A total of 4 × 10^5^ were plated onto each well of a 6-well plate to form a cell monolayer for 24 h. Next, a straight scratch was created using 100 µl yellow tip in each well, and next cells were incubated with treatment protocol for 48 h. The healing rate or migration of cells was observed at interval points 0, 24 h and 48 h of treatment and images were obtained using light microscopy (IM-3/ OPTIKA) and CCD camera (TrueChrome II). The closure or wound healing rate was calculated using Image J software (ver. 1.44p) using the below formula: healing rate (percentage) = (New scratch area - second scratch area)/ New scratch area × 100.

### Statistical analysis

The statistical analysis was done using the student’s t-test, and ANOVA as applicable, followed by Tukey post hoc test (GraphPad Software, ver. 9.0.1). A value of *P* < 0.05 was reflected as statistically significant between different groups. Data were mean ± S.D. of independent triplicate experiments. In the figures, statistical significance was shown as * *P* < 0.05.

## Results

**Fig. 1:**
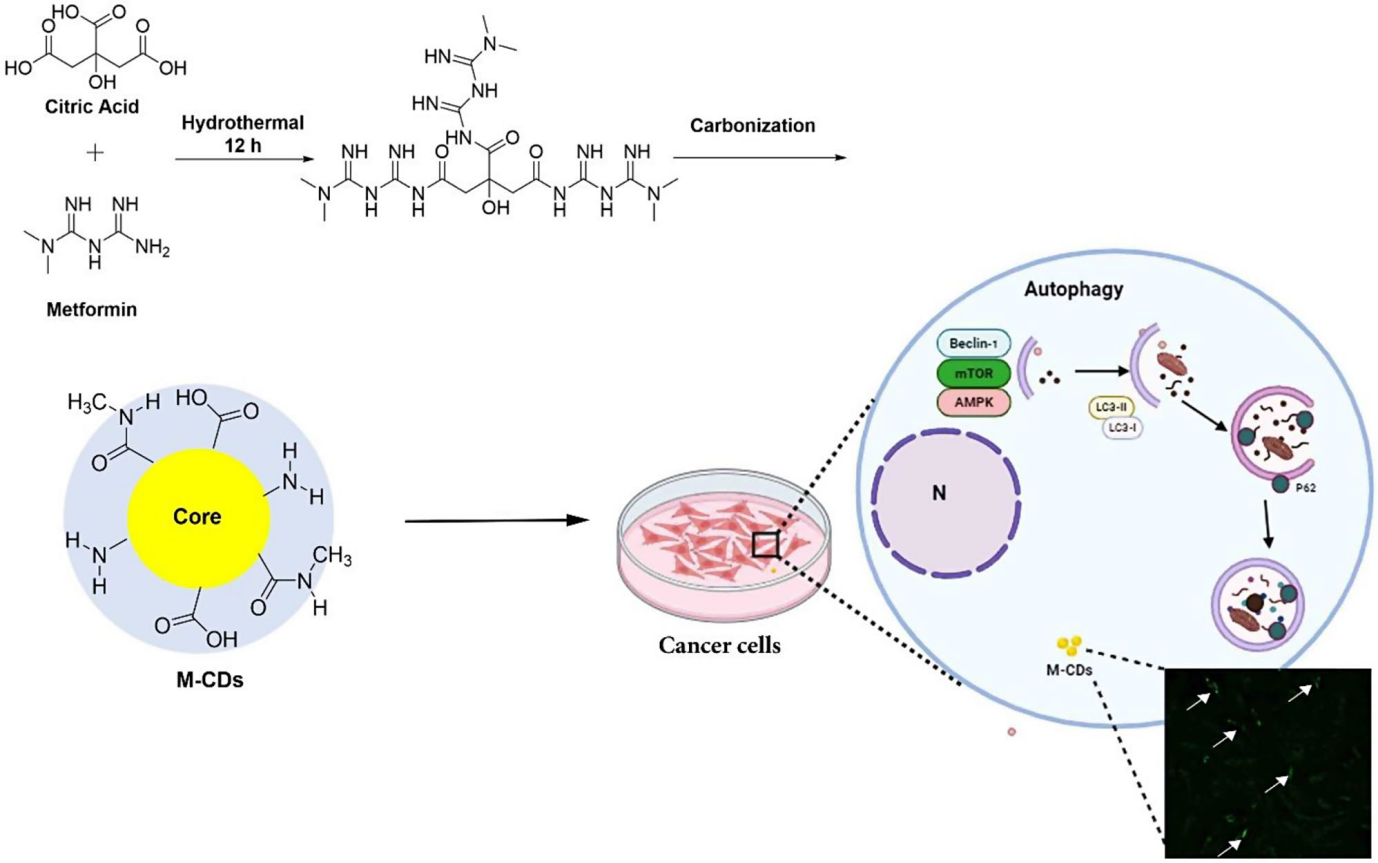
Schematic representation of the possible synthetic route of M-CDs by hydrothermal method

As can be seen in Fig. 1, a simple one-step hydrothermal/carbonization reaction was carried out between CA and metformin to prepare M-CDs. On the other hand, at the beginning of the reaction, a simple condensation occurred between the amine groups of metformin and terminal carboxylic moeites of CA, leading to the formation of a polyamide structure. Then, during the heating process, carbonization of the resulting polymers was carried out and resulted in M-CDs product.

**Fig. 2:**
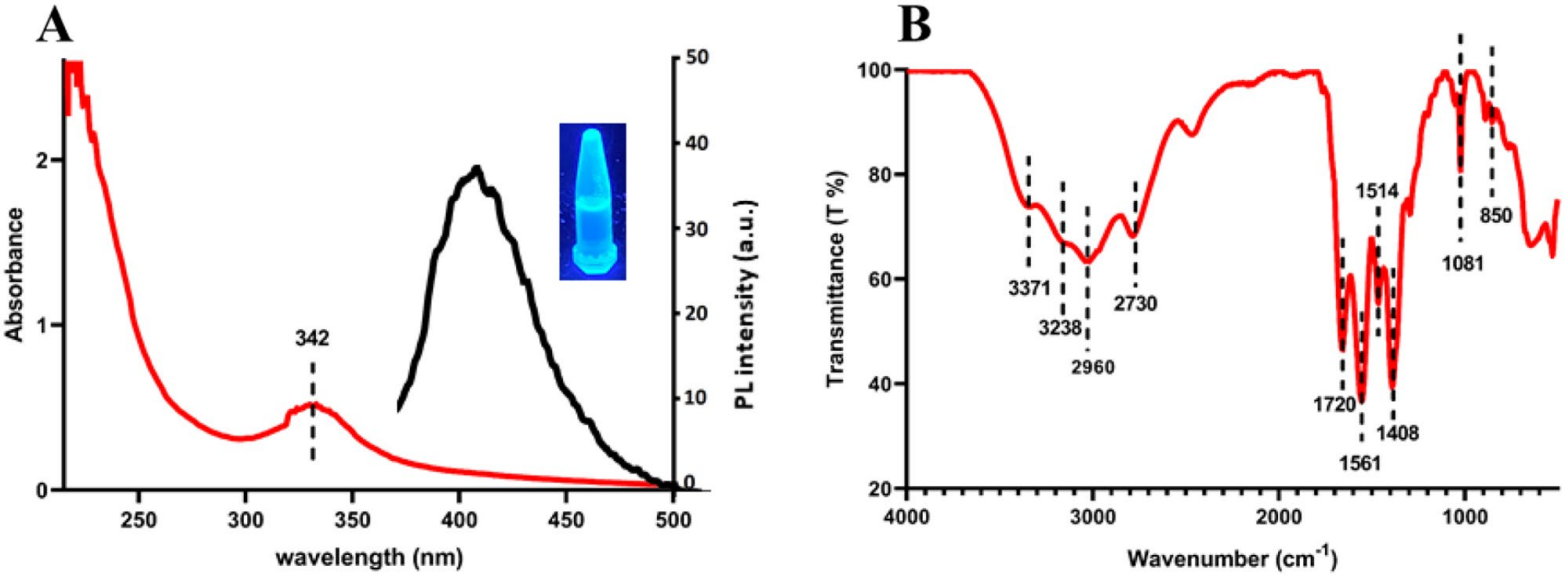
**A**) UV-Vis absorption (red line), PL spectra (black line) and **B**) FTIR spectrum of M-CDs

UV-vis spectrum of M-CDs showed a moderate characteristic adsorption band at 342 nm (Fig. 2A, red line). This band was also reported by previous studies which explained n-π* electronic transition of C = N and C-N functional groups in all N-doped CDs [17, 18]. Additionally, blue fluorescence with λ_max_ at 420 nm under 360 nm UV light could be seen in PL spectrum of M-CDs (Fig. 2A, black line). The successful formation of the chemical bonds and the related functional groups on the structure of M-CDs were elucidated from FTIR spectra. The characteristic peaks at 3371 cm^− 1^ and 3238 cm^− 1^ are attributed to the stretching vibration of N-H and O-H, respectively. Peaks at 2960 cm^− 1^ and 2730 cm^− 1^ corresponded to C-H stretching vibration. The peaks at the lower wavenumber region, namely 850 cm^− 1^, 1081 cm^− 1^, 1408 cm^− 1^, 1514 cm^− 1^, 1561 cm^− 1^ and 1720 cm^− 1^ were assigned to N-H stretching, C-N stretching, O-H bending, N-O stretching, N-H bending and C = O stretching, respectively. It should be highlighted that there is no N-O bond in the starting materials structures but the existence of the N-O bond could be found in M-CDs FTIR spectra (Fig. 2B).

**Fig. 3:**
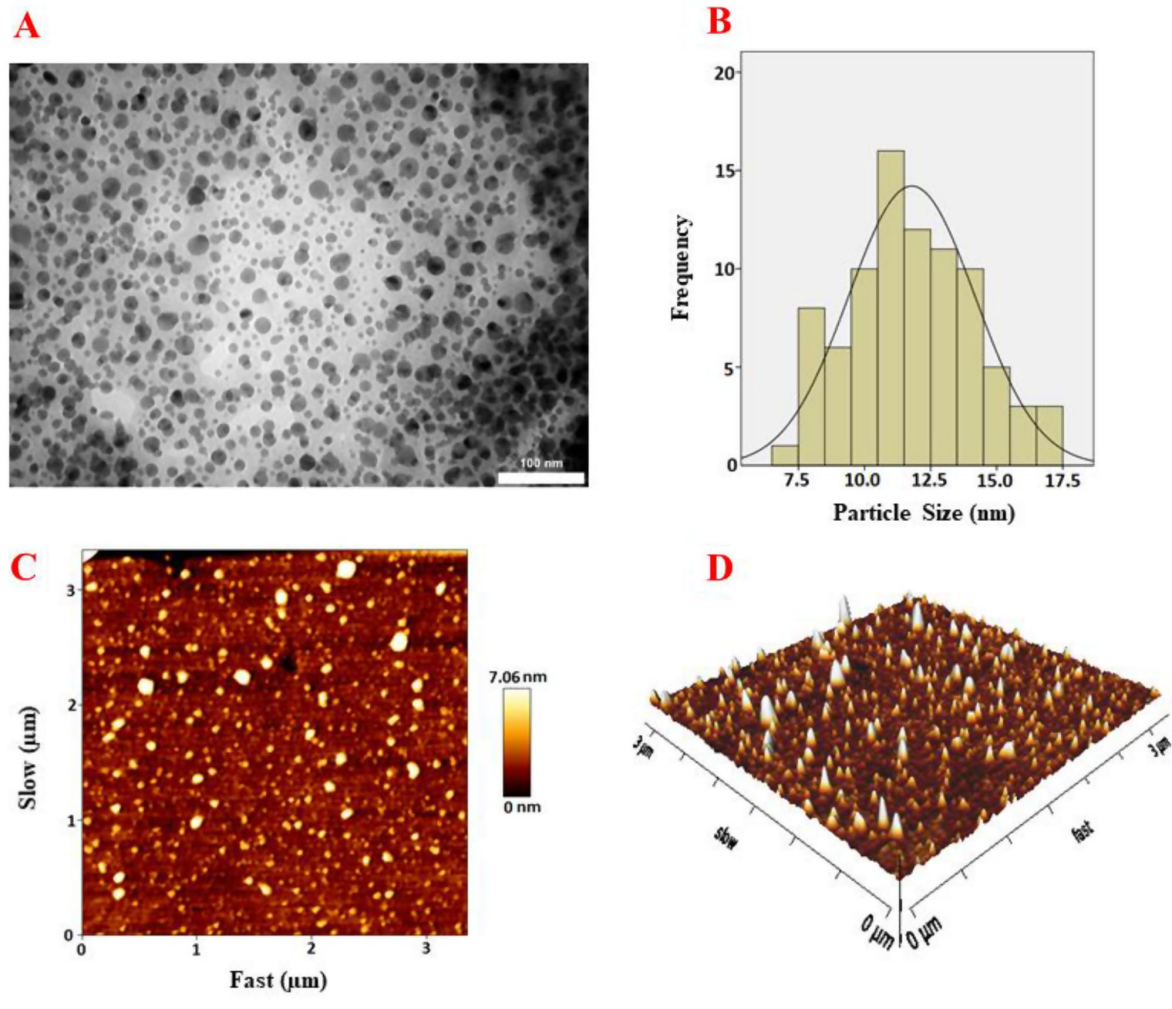
**A**) TEM image, **B**) size distribution histogram, **C**) AFM topography image and **D**) AFM 3D image of the as-prepared M-CDs

Based on Fig. 3A and B, The TEM image illustrated that M-CDs had a mono-dispersed spherical particle with an avarage diameter of carbon dots of about 9.5 nm. Moreover, the AFM topography image and 3D image (Fig. 3C and D) indicated the spherical shape of M-CDs, which was in good agreement with TEM results.

### Metformin and M-CDs decreased the viability of cancer cells

**Fig. 4:**
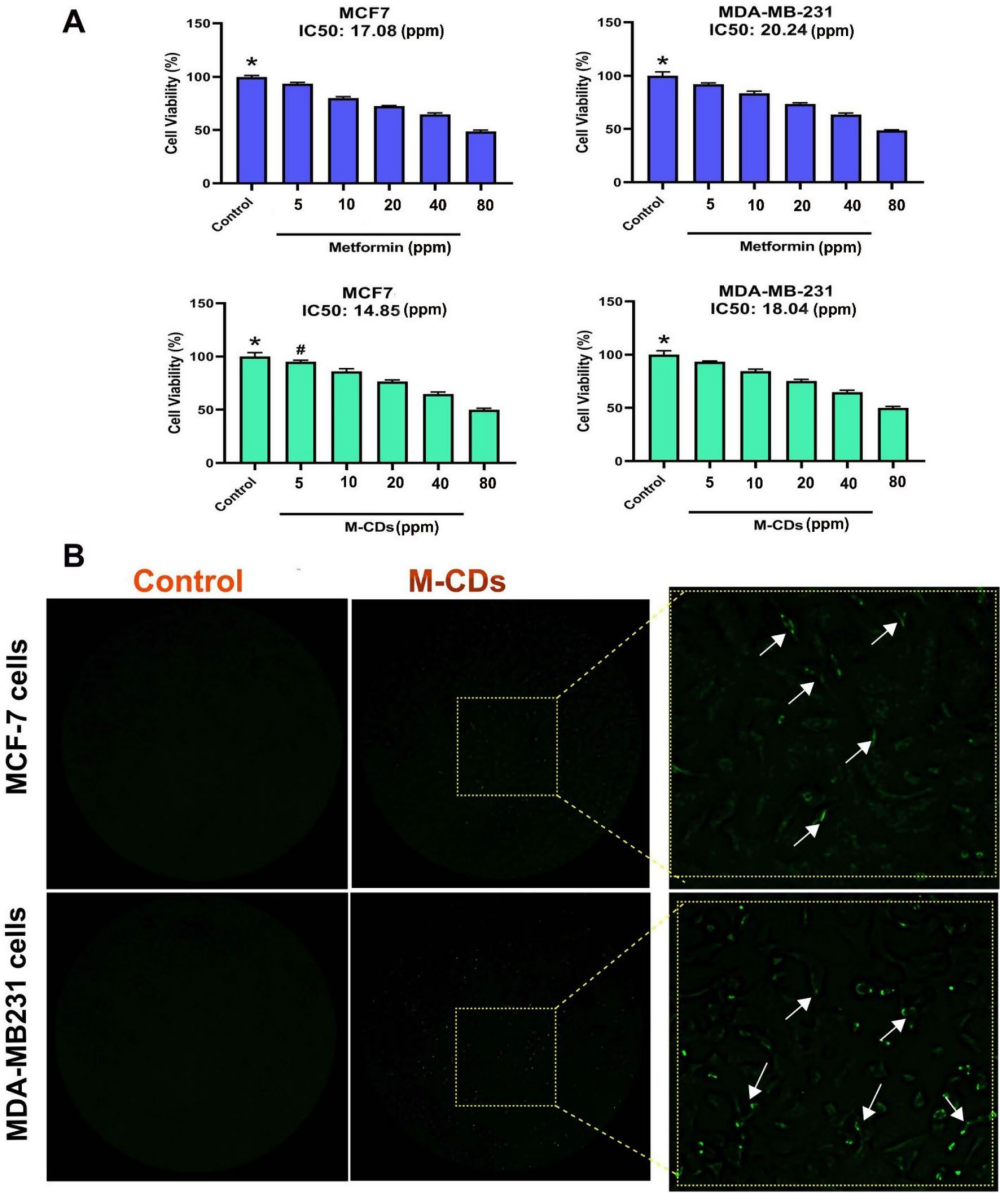
MTT assay for cell viability of MCF-7 and MDA-MB-231 cells treated with metformin and CDs from metformin (M-CDs) for 24 (**A**). IC50 for each treatment has been specified on relevant images. Uptake of M-CDs by MCF-7 and MDA-MB-231 cells performed by immunofluorescence microscopy (**B**). Arrows show M-CDs were captured by both cancer cells. ANOVA and Tukey post hoc test. Data were mean ± S.D. of independent triplicate experiments. Statistical significance was shown as * *P* < 0.05; ^#^*P* > 0.05 between the control and other groups. Magnification is × 10

To investigate the possible cytotoxic effect of metformin and M-CDs on breast cancer cells, we used communal MTT assay throughout 24 h and 48 h. Results showed that metformin and M-CDs decreased the viability of both MCF-7 and MDA-MB-231 cells compared to control cells either 24 or 48 h post-treatment, representing the cytotoxic activity of these compounds on cancer cells (Fig. 4A). Furthermore, we found that IC50 of metformin for MCF-7 cells and MDA-MB-231 cells were 17.08 ppm and 20.24 ppm respectively. IC50 of M-CDs for the same cells were 14.85 ppm and 18.04 ppm, respectively. In addition, as seen in the next sections, M-CDs induced cell death by increasing the protein level of Caspase-3 in cancer cells. These data indicate that M-CDs significantly decreased the viability of breast cancer cells.

### Cancer cells could uptake M-CDs

As shown by Fig. 4B, both cancer cells successfully captured M-CDs. These particles have a size between 10 and 20 nm that can easily enter into cells.

### Cell morphological assay

**Fig. 5:**
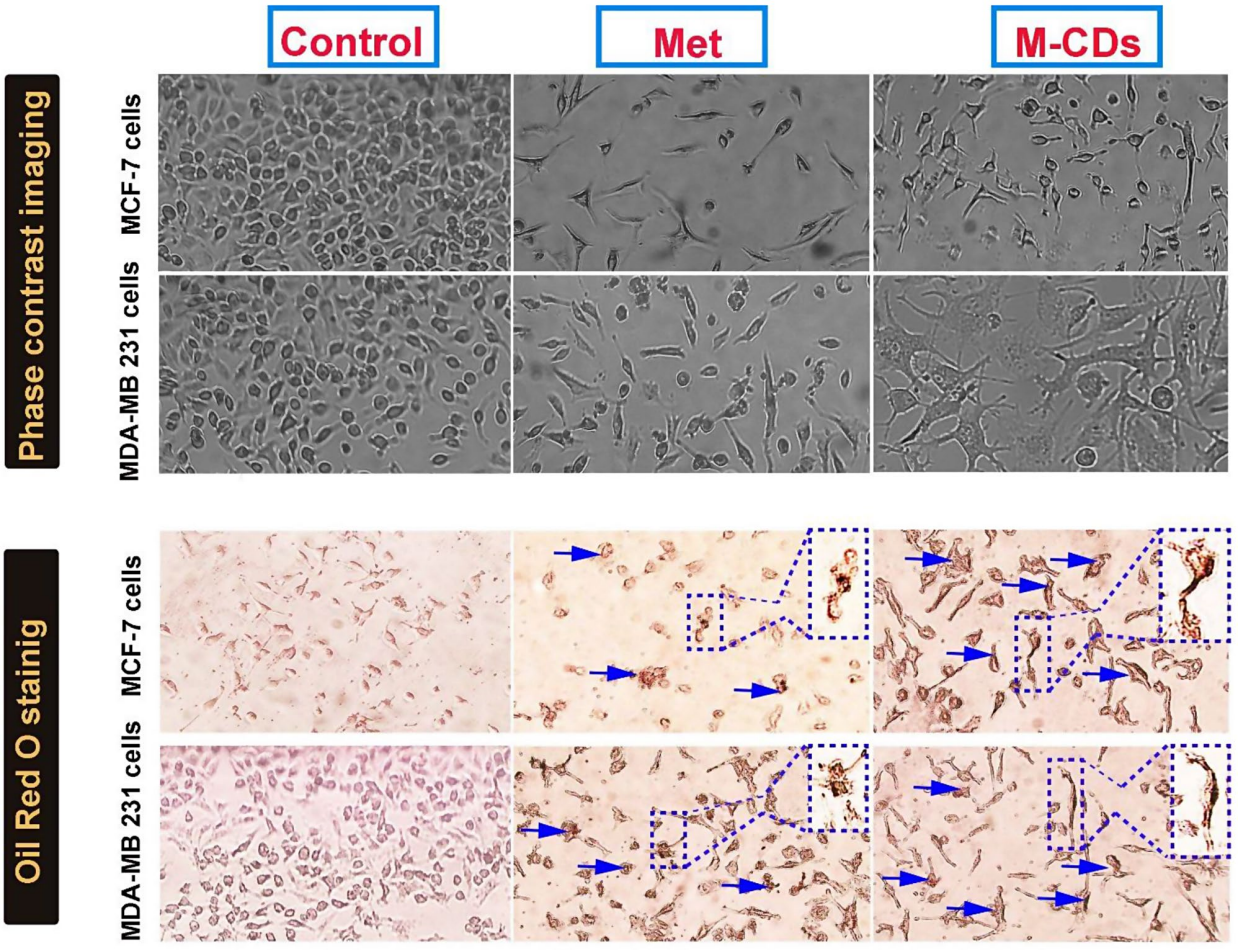
Representative images of MCF-7 and MDA-MB-231 cells treated with metformin (Met) and CDs from metformin (M-CDs) for 24. Phase contrast imaging (upper) and Oil Red Oil staining (lower). Blue arrows show Oil Red O positive cells. Magnification is × 10

As we observed the intracellular distribution of M-CDs, we hypothesized M-CDs may cause cellular morphological alterations. Then, we found morphological changes in the treated cells by a simple phase contrast microscopy (Fig. 5A). M-CDs induced some morphological changes such as abnormal cell processes, and irregular and flattened shapes, especially in M-CDs treated MDA-MB-231 cells.

### M-CDs increased Oil Red O positive cells

To investigate cellular toxicity, we performed an Oil Red O staining assay. As shown by Fig. 5B, the number of Oil Red O positive cells was increased in M-CDs treated cells.

### Production of intracellular ROS increased in treated cells

**Fig. 6:**
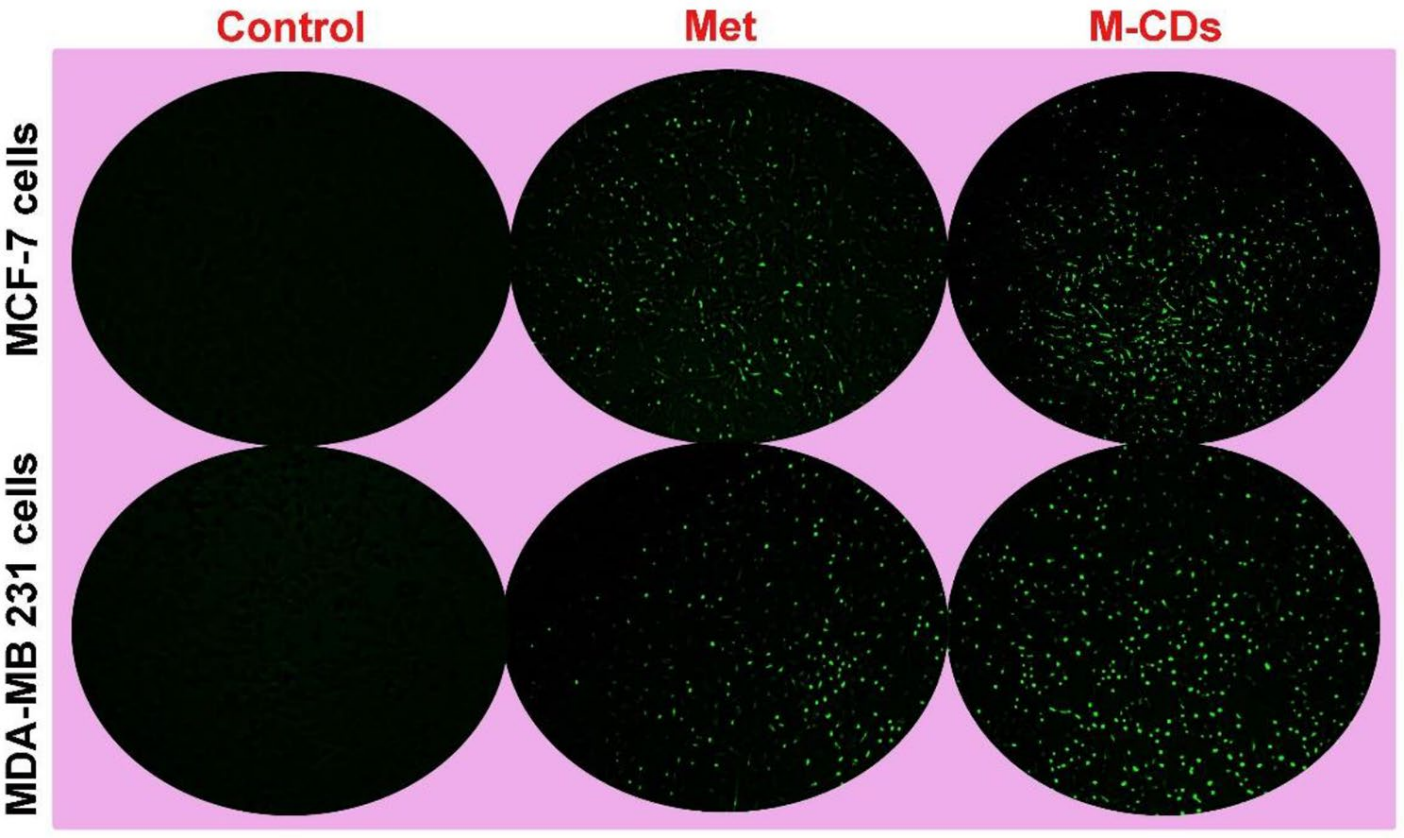
Immunofluorescence microscopy imaging for ROS production by MCF-7 and MDA-MB-231 cells treated with metformin (Met) and CDs from metformin (M-CDs) for 24. Magnification is × 10

To discover the cellular cytotoxicity triggered by M-CDs, we assessed the production of ROS in cancer cells. As shown in Fig. 6, we observed that cells treated with metformin and M-CDs showed a high intensity of green fluorescence, representing cellular oxidative stress, which may be due to increased ROS inside cancer cells. At first sight, M-CDs profoundly caused ROS generation owing to damage to cellular organelles such as mitochondria.

### Autophagy flux was initiated in treated cells

**Fig. 7:**
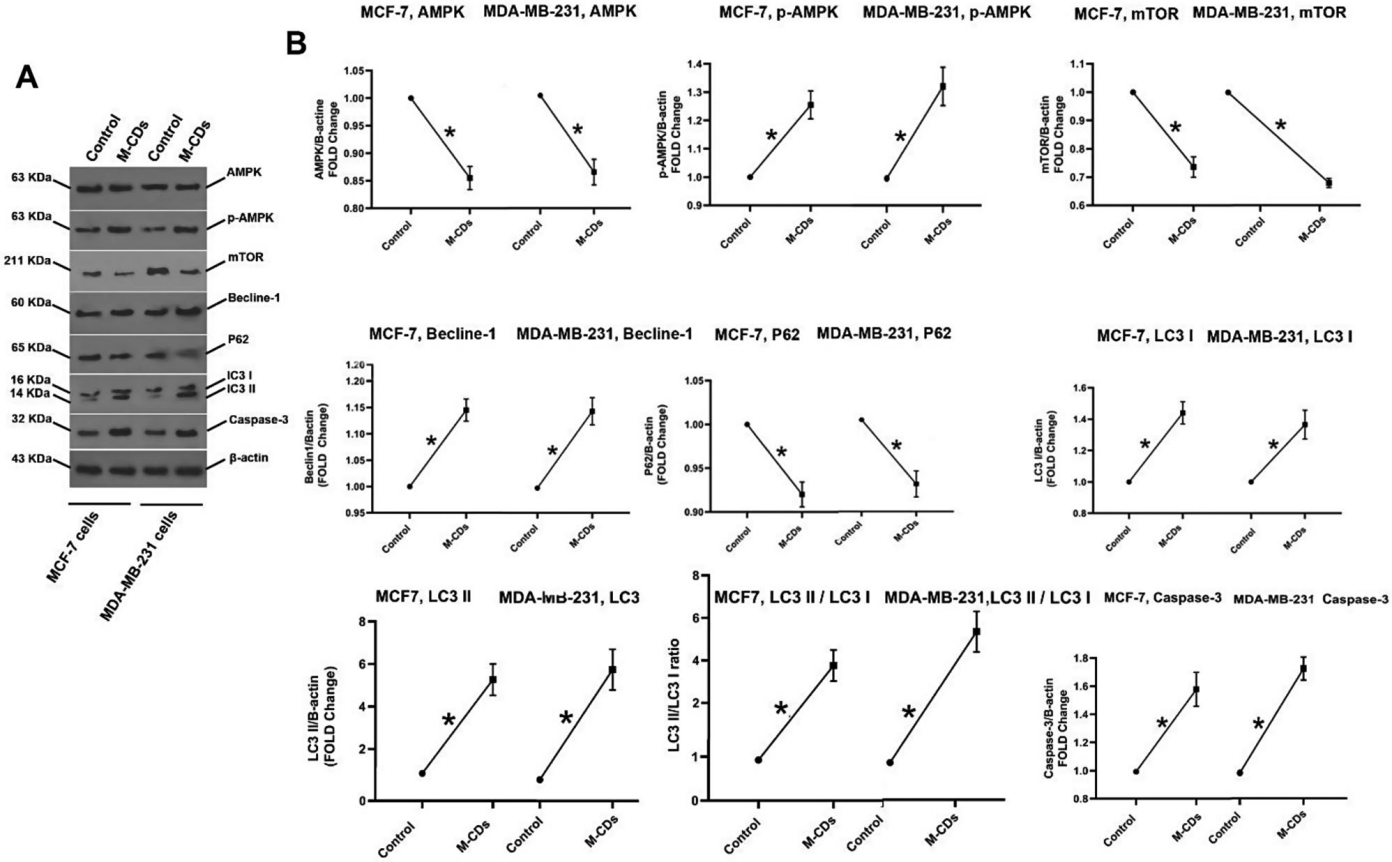
Western blotting analysis for autophagic and apoptotic proteins (**A**). Analysis for proteins band of western blotting (**B**). T’-test used for analysis. Data were mean ± S.D. of independent triplicate experiments. Statistical significance was shown as * *P* < 0.05 between the control and treated cells

We also investigated the autophagy signaling pathway in cells via western blotting. Our results showed that autophagy flux was initiated in treated cells. For example, we found that protein levels of AMPK were decreased, whereas phosphorylation of AMPK (p-AMPK) was increased in both M-CDs treated cells (*p* < 0.05; Fig. 7A, B). The level of mTOR protein was decreased in treated cells (*p* < 0.05). We also found that the levels of Becline-1, LC3 I, LC3 II, and the LC3 II/ LC3 I ratio were decreased, while the protein level of P62 was decreased in treated cells compared to the control cells (*p* < 0.05; Fig. 7A, B).

### Protein levels of Caspase-3 were increased in treated cells

Our results from western blotting showed that expression of Caspase-3, a main executioner of apoptosis, was up-regulated in M-CDs treated cells (*p* < 0.05, Fig. 7A, B), representing an apoptosis induction.

### The wound healing rate of M-CDs treated cells was decreased

**Fig. 8:**
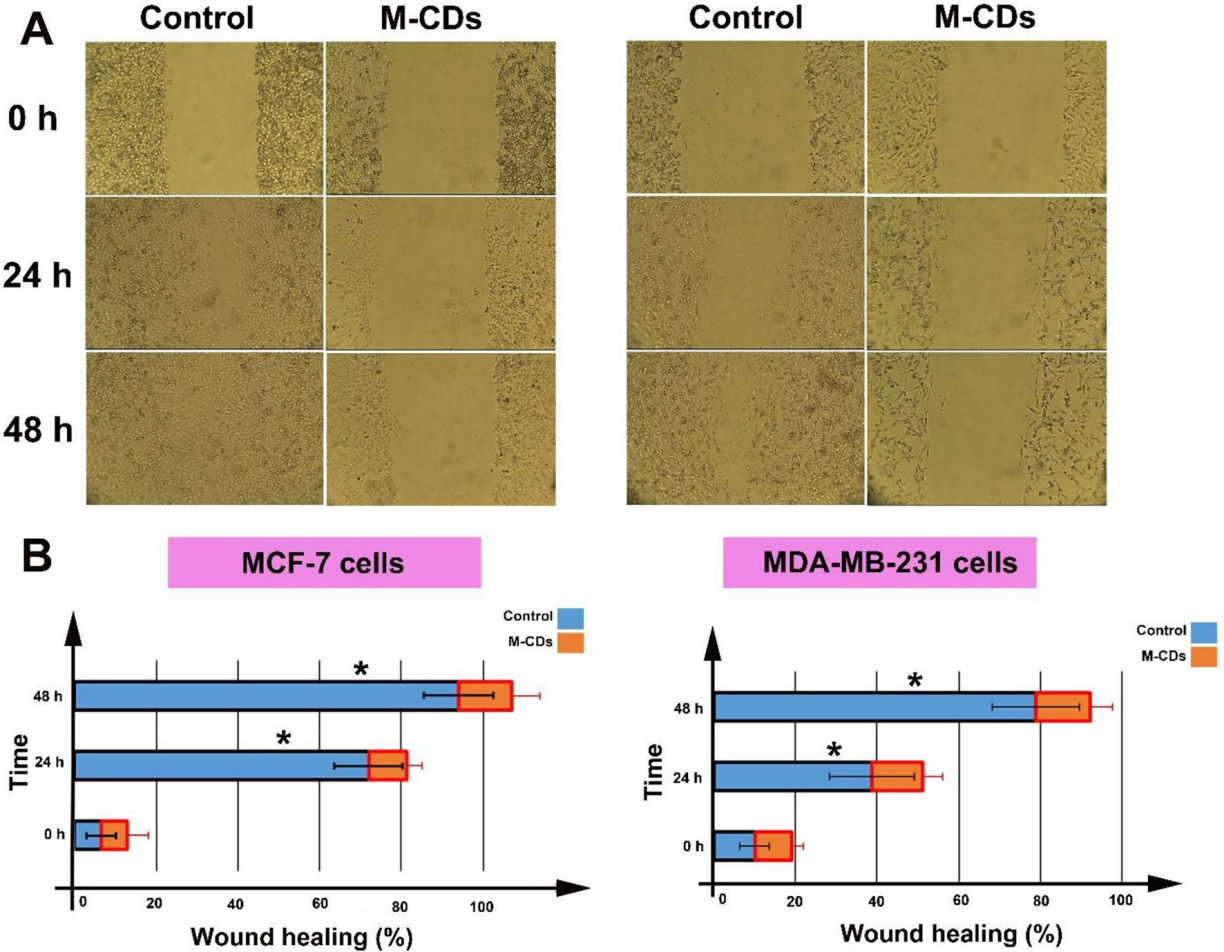
Wound healing rate of MCF-7 and MDA-MB-231 cells (**A** and **B**). T’-test used for analysis. Data were mean ± S.D. of independent triplicate experiments. Statistical significance was shown as * *P* < 0.05 between control and treated cells. Magnification is × 10

In vitro, the scratch assay was used to investigate the effect of M-CDs on the wound healing rate and migration potential of tumor cells. As shown in Fig. 8, we observed that the wound healing rate of cells treated with M-CDs was significantly decreased throughout 24 h and 48 h as compared to the control group. In MCF-7 cells, the percentage of wound healing of the control group and M-CDs group were 94.1 ± 8.5% and 13 ± 7.08% for 48 h, respectively (*p* < 0.05, Fig. 8A and B). The same results were obtained for MDA-MB-231 cells, for example, for 48 h, the wound healing rates were 79 ± 10.72% and 13.25 ± 5.3% for control and M-CDs treated cells, respectively (*p* < 0.05, Fig. 8A and B).

## Discussion

During the last two decades, substantial attention has been devoted to the biomedical applications of CDs due to their high water dispensability, excellent photoluminescence, good biocompatibility and low toxicity. We aimed to prepare CDs from metformin and investigate the impact of CDs on two human breast cancer cells MCF-7 and MDA-MB cells, and also at the same time study autophagy and apoptosis pathways in these cells.

We first found that M-CDs caused profound cytotoxicity, initiating low cell viability against control cells even metformin-treated cells. These data support previous findings in the literature that indicated metformin caused a reduction in cell viability in different cells [19, 20]. Our results have several similarities with previous findings, which indicated that CDs can reduce the viability of cells and induce cell death [21, 22]. Furthermore, we found that IC50 of metformin for MCF-7 cells and MDA-MB-231 cells were 17.08 ppm and 20.24 ppm respectively after 24 h. IC50 of M-CDs for the same cells were 14.85 ppm and 18.04 ppm, respectively after 48 h. This would appear to indicate that M-CDs are more cytotoxic than metformin and thus may show lower systemic toxicity [23]. In addition, as seen in the next sections, M-CDs induced cell death by increasing the protein level of Caspase-3 in these cells. As shown by Fig. 4B, both cancer cells successfully captured M-CDs. These particles have a size between 10 and 20 nm that can easily enter into cells. Our results have several similarities with Cilingir et al.’s findings that M-CDs were captured by HEK-293 human embryonic kidney and glioblastoma cell lines and localized in mitochondria [24]. Previous studies have indicated that CDs can be uptake by cells and distributed in the cytoplasm, which makes them a new tool for cell tracking experiments [25, 26]. Mitochondrial localization is an exceptional property for several types of CDs since mitochondria play a noteworthy role in different cellular processes, for example, producing ATP and ROS, intracellular Ca2 + signaling, regulation, regulation of cellular redox, and launch apoptosis and autophagy, and cellular homeostasis as a critical energy-producing organelle within healthy and cancer cells [27, 28]. As we observed the intracellular distribution of M-CDs, we hypothesized that M-CDs may cause cellular morphological alterations. Then, we found morphological changes in the treated cells by a simple phase contrast microscopy (Fig. 5A). M-CDs induced some morphological changes such as abnormal cell processes, and irregular and flattened shapes, especially in M-CDs treated MDA-MB-231 cells, proposing cellular senescence. On the other hand, the number of Oil Red O positive cells was increased in M-CDs treated cells. This observation may correlate with the accumulation of lipid droplets within the cytoplasm, suggesting induction of apoptosis. Our more recent study showed that CDs from Saffron caused lipotoxicity and increased the number of cells stained with Oil Red O solution [29, 30]. In our opinion, M-CDs may localize in mitochondria that interfere with the lipid biosynthesis pathway, accumulating lipid droplets within the cytoplasm [24, 31]. As shown in Fig. 6, we observed that cells treated with metformin and M-CDs showed a high intensity of green fluorescence, representing cellular oxidative stress, which may be due to increased ROS inside cancer cells. At first sight, M-CDs profoundly caused ROS generation owing to damage to cellular organelles such as mitochondria. Our results land similar to previous studies that described CDs could increase intracellular ROS, causing oxidative stress, and cell death [32, 33]. Therefore, CDs-induced cytotoxicity may be caused by ROS generation and organelle damage. In this context, we belive that accumulation of M-CDs inside cells, caused ROS production, morphological changes and finally induction of apotosis, which decrease cell viability.

In the next panel, our results showed that autophagy flux was initiated in treated cells. For example, we found that protein levels of AMPK were decreased, whereas phosphorylation of AMPK (p-AMPK) was increased in both M-CDs treated cells. In the autophagy pathway, p-AMPK (activated form) inhibits mTOR, which inhibits ULK-1 complex (autophagy initiation complex) [34]. Other evidence for autophagy flux comes from the results of mTOR and Becline-1 analysis that shows protein levels of total mTOR decreased while protein levels of Becline-1 were increased in M-CDs treated cells, suggesting autophagy flux. In addition, we found that protein levels of P62 and the LC3 II/ I ratio were down-regulated and increased in M-CDs treated cells, respectively. In the autophagy pathway, LC3-I is converted to LC3-II and engaged in the membrane of the developing phagophore, a double membrane necessary for the recycling of damaged organelles and proteins [34]. P62 is a multifunctional protein with a role in clearing protein aggregates. When autophagy is inhibited P62 accumulates but when autophagy is prompted, the P62 level is decreased [34]. These results indicated that autophagy flux was activated in cells because an increase in upstream proteins such as p-AMPK and Becline-1, and a change in downstream proteins such as P62, LC3 I, and LC3 II are signs of the formation of autophagosomes and autolysosomes in treated cancer cells [34, 35]. Similar to our finding, Hua et al. declared that graphene-based CDs promoted autophagy in SGC-7901 cells through the mTOR signaling pathway [36]. More recently, researchers produced manganese-doped graphene CDs for anticancer assays. They found that these CDs could promote excessive autophagy and induce apoptosis in HepG2 cancer cells [37]. Thus far, the dual role of autophagy both in tumor progress and suppression remains debatable [38].

Although we did not assay mitophagy, however to some extent, we think damage to mitochondria may contribute to inducing autophagy flux and autolysosome formation. It can thus be suggested that M-CDs may accumulate in mitochondria and increase ROS production, and finally organelles damage; such events initiate autophagy flux in cells. Our results from western blotting showed that expression of Caspase-3, a main executioner of apoptosis, was up-regulated in M-CDs treated cells, representing an apoptosis induction. Similarly, cadmium telluride CDs have been shown to increase cellular toxicity in human neuroblastoma (SH-SY5Y) cells and induce apoptosis [39]. More recently, a study carried out with Ku and co-workers confirmed that graphene quantum dots (GQDs) promoted cell cycle arrest and apoptosis in four lines of breast cancer cells [40]. Our results may show a correlation to data from MTT and ROS assays that treated cancer cells had low levels of viability and high levels of ROS production. Furthermore, the antiproliferative effect of M-CDs cannot be entirely enlightened by ROS generation as the autophagy pathway may crosstalk to the apoptosis pathway. In a study, using Cadmium-based CDs, Fan et al. declared that autophagic flux and autophagic cell death were promoted by these CDs, which clarified the key role of autophagy in Cadmium-based CDs induced toxicity [41]. It is generally accepted that autophagy can also activate apoptosis under certain situations via the stimulation of Caspase-8 and the lessening of apoptosis inhibitors [42, 43]. Our findings would seem to show that ROS production and autophagy elements trigger activation of Caspase-3, and are finally complemented by the characteristics of apoptosis [42]. To the best of our knowledge, this is the first finding that M-CDs through associated regulation of autophagy and apoptosis favor the removal of both breast tumor cells. Therefore, further studies, which take the autophagy-apoptosis relationship into account, will need to be undertaken.

We also investigated the effect of M-CDs on the wound healing rate and migration potential of tumor cells. We observed that the wound healing rate of cells treated with M-CDs was significantly decreased throughout 24 h and 48 h as compared to the control group. These results may explain a reduction in the migration capacity and mobility of cancer cells [44]. To the best of our knowledge, this is the first report and further scrutiny may uncover the mechanisms that inhibit the migration rate of cells. In addition, by involving normal cells (non-cancerous cells), further study is needed to confirm our results.

## Conclusions

In this study, for the first, M-CDs were synthesized by simple one-pot hydrothermal treatment without further purification. The successful formation of M-CDs was confirmed using various techniques namely Uv-Vis, PL, FTIR, TEM, and AFM. Our results indicated that M-CDs were more cytotoxic and could be uptaken by cancer cells. These particles caused morphological changes and inhibited cell viability. M-CDs accumulated inside cells and caused lipotoxicity and ROS production. In addition, M-CDs activated autophagy flux and increased apoptosis in cells. We proposed that M-CDs can be tracked inside cells, which caused cellular toxicity, induced autophagy flux, and increased apoptosis rate.

## Data Availability

No datasets were generated or analysed during the current study.

## Abbreviations

QD: Quantum dot
CDs: Carbon dots
M-CDs: CDs-derived from metformin
FTIR: Fourier transform infrared
PL: Photoluminescence
TEM: Transmission electron microscopy
AFM: Atomic force microscopy
FBS: Fetal bovine serum
MTT: 3-[4,5-dimethylthiazol-2yl]-2,5-diphenyl-tetrazolium bromide
DMSO: Dimethyl sulfoxide
PFA: Paraformaldehyde
